# Supplementary material for: Myelin-Weighted Imaging Presents Reduced Apparent Myelin Water in Patients with Alzheimer’s Disease
Source: Diagnostics (Basel). 2022 Feb 9;12(2):446. doi: 10.3390/diagnostics12020446 (PMC8871299; doi:10.3390/diagnostics12020446)
Supplement: Supplementary file 1 [file diagnostics-12-00446-s001.zip › diagnostics-1544684-supplementary.pdf]

# Supplementary Tables

**Table S1. Results of the voxel-based comparisons of apparent MWF among the three participant groups.**

| Group analysis | Cluster size | Cluster location | BA | Talairach coordinates | Z score |
|---|---|---|---|---|---|
| **CN>AD** | 259548 | Rt Limbic Posterior Congulate | | 5.52, -52.25, 2014 | 65535 |
| | | Lt Parietal Precuneus | | -14.17, -66.42, 36.03 | 7.88 |
| | | Lt Parietal Supramarginal Gyrus | | -44.59, -53.04, 30.03 | 7.171 |
| **MCI>AD** | 154712 | Lt Sub-lobar Insula GM | 13 | -36.06, -35.13, 19.71 | 6.777 |
| | | Lt Temporal Sub-Gyral | | -37.34, -56.31, 5.52 | 6.765 |
| | | Lt Limbic Posterior Cingulate | | -12.43, -48.71, 13.42 | 6.652 |
| | 4600 | Rt Pons | | -9.08, -19.09, -30.71 | 4.983 |
| | | Rt Posterior Cerebellar Tonsil GM | | 25.76, -43.68, -39.51 | 4.358 |
| | | | | 14.75, -27.85, -42.25 | 4.244 |
| | 548 | Lt Posterior Cerebellar Tonsil GM | | -28.38, -44.79, -40.53 | 3.998 |

This table is listed in the result of the voxel-based full factorial ANCOVA test of apparent myelin water fraction (MWF) maps among the three subject groups. Participant's age was used as covariate. We did not find any significant differences of apparent MWF values for the following tests: CN <AD, CN>MCI and CN <MCI, and MCI<AD.

CN, cognitively normal; MCI, mild cognitive impairment; AD, Alzheimer’s disease; Rt, right; Lt, left; BA, Brodmann area

**Table S2. Results of the voxel-based comparisons of GMV among the three participant groups**

| Group analysis | Cluster size | Cluster location | BA | Talairach coordinates | Z score |
|---|---|---|---|---|---|
| **CN>MCI** | | | | | |
| | 789 | Rt limbic cingulate gyrus | 24 | 4.04, -17.77, 37.8 | 6.267 |
| | | Rt frontal paracentral lobule | 31 | 5.32, -26.81, 43.72 | 4.722 |
| | 2664 | Rt temporal fusiform fyrus | 20 | 43.73, 0.49, -20.61 | 6.225 |
| | | Rt temporal middle temporal gyrus | 21 | 49.22, -5.53, .17.03 | 5.941 |
| | | Rt temporal inferior temporal gyrus | 20 | 53.44, -10.61, -22.85 | 5.165 |
| | 917 | Rt limbic uncus | 38 | 23.1, 4.69, -34.07 | 5.501 |
| | 201 | Rt frontal superior frontal gyrus | 9 | 11.48, 55.81, 20.57 | 5.038 |
| | 271 | Lt temporal superior temporal gyrus | 38 | -39.5, 6.77, -24.13 | 5.028 |
| | 248 | Rt cerebellum anterior lobe culmen | | 7.17, -67.23, -4.67 | 5.011 |
| | | Rt occipital lingual gyrus | 19 | 12.68, -63.45, -0.17 | 4.803 |
| | 355 | Rt limbic parahippocampal gyrus | | 26.98, -17.99, -18.59 | 4.933 |
| | 105 | Rt frontal middle frontal gyrus | 6 | 24.67, -12.46, 54.86 | 4.600 |
| | | Rt frontal precentral gyrus | 6 | 24.72, -17.52, 48.98 | 4.536 |
| **CN>AD** | | | | | |
| | 180927 | Rt sub-lobar claustrum | | 37.93, -16.43, -6.1 | 65535 |
| | | Rt limbic parahippocampal gyrus | 28 | 24.2, -17.97, -18.64 | 65535 |
| | | Rt temporal middle temporal gyrus | 21 | 50.59, -7.06, -15.81 | 65535 |
| | 726 | Rt cerebellum posterior pyramis | | 31.14, -72.86, -34.53 | 3.971 |
| | | Rt cerebellum posterior uvula | | 30.99, -76.7, -24.09 | 3.480 |
| | 454 | Lt cerebellum posterior inferior semi-lunar lobule | | -27.12, -70.9, -38.03 | 3.864 |
| | | Lt cerebellum posterior cerebellar tonsil | | -38.09, -52.02, -43.19 | 3.549 |
| **MCI>AD** | | | | | |
| | 18772 | Rt limbic parahippocampal gyrus | 28 | 21.41, -18.09, .17.34 | 6.264 |
| | | Rt sub-lobar claustrum | | 37.93, -16.43, -6.1 | 6.093 |
| | | Rt sub-lobar lentiform nucleus | | 31.05, -6.35, -7.96 | 6.018 |
| | 1644 | Lt frontal medial frontal gyrus | 10 | -13.23, 36.07, -7.39 | 5.461 |
| | | Rt limbic anterior cingulate | 24 | 6.14, 31.38, -3.46 | 5.049 |
| | | Lt frontal middle frontal gyrus | 11 | -21.58, 38.64, -4.59 | 4.976 |
| | 477 | Rt limbic parahippocampal gyrus | 30 | 11.29, -37.42, 7.68 | 5.276 |
| | 3296 | Lt sub-lobar insula | | -31.47, 19.61, 2.9 | 5.224 |
| | | Lt temporal sub-gyral | 21 | -39.77, -10.3, -8.18 | 5.062 |
| | | Lt frontal precentral gyrus | 44 | -41.29, 7.96, 7.04 | 4.898 |
| | 183 | Lt limbic parahippocampal gyrus | 36 | -36.99, -29.49, -14.01 | 5.048 |
| | | Lt temporal fusiform gyrus | 20 | -45.36, 31.1, -11.6 | 4.889 |
| | 262 | Lt temporal middle temporal gyrus | 21 | -56.41, -18.34, -11.93 | 4.834 |
| | 256 | Lt temporal middle temporal gyrus | 22 | -47.07, -40.3, 10.47 | 4.789 |
| | | Lt temporal superior temporal gyrus | 22 | -46.98, 32.79, 5.78 | 4.281 |
| | 504 | Lt cerebellum posterior cerebellar tonsil | | -35.3, -54.7, -44.74 | 4.646 |
| | | Lt cerebellum posterior inferior semi-lunar lobule | | -34.01, -65.01, -40.29 | 4.077 |
| | 182 | Rt limbic anterior cingulate | 10,32 | 11.75, 50.92, -1.51 | 4.569 |
| | 181 | Lt limbic parahippocampal gyrus | 35 | -23.11, -29.56, -13.78 | 4.449 |
| | 114 | Lt occipital cuneus | 18 | -11.2, -68.22, 20.6 | 4.274 |

Subject's total intracranial volume (TIV), age, and gender were used as covariates. The gray matter volume (GMV) was not significantly different to the following tests: CN<AD, CN<MCI, and MCI<AD.

CN, cognitively normal; MCI, mild cognitive impairment; AD, Alzheimer’s disease; Rt, right; Lt, left; BA, Brodmann area

**Table S3. Results of the voxel-based comparisons of WMV among the three participant groups**

| Group analysis | Cluster size | Cluster location | BA | Talairach coordinates | Z score |
|---|---|---|---|---|---|
| **CN>MCI** | | | | | |
| | 16133 | Lt parietal precuneus | | -18.37, -54.77, 42.02 | 6.892 |
| | | Rt limbic cingulate gyrus | | 8.21, -20.45, 36.26 | 6.124 |
| | 5899 | Rt temporal sub-gyral | | 40.59, -26.87, -0.28 | 6.031 |
| | | Rt frontal inferior frontal gyrus | 47 | 25.66, 8.57, -16.1 | 5.190 |
| | 5735 | Lt frontal sub-gyral | | -34.49, 24.66, 23.6 | 5.826 |
| | | Rt sub-lobar extra nuclear | | 6.08, 21.34, -1.71 | 5.097 |
| | | Lt frontal sub-gyral | | -30.13, 26.07, 8.94 | 5.040 |
| | 415 | Rt temporal superior temporal gyrus | | 43.02, -47.28, 20.8 | 5.735 |
| | 436 | Rt occipital lobe | | 12.73-.67.26, -4.58 | 5.345 |
| | 3564 | Lt temporal sub-gyral | | -36.74, 1.16, -24.61 | 5.141 |
| | | Lt limbic parahippocampal gyrus | | -37, -24.03, -12.14 | 5.137 |
| | 498 | Rt sub-lobar extra-nuclear | | 29.5, 18.51, 11.94 | 5.126 |
| | | Rt frontal sub-gyral | | 33.54, 23.04, 23.24 | 4.940 |
| | 496 | Rt frontal medial frontal gyrus | | 14.29, 53.27, 17.67 | 4.913 |
| | 290 | Rt parietal precuneus | | 13.59, -56.07, 39.73 | 4.634 |
| | 106 | Rt frontal superior frontal gyrus | | 20.73, 15.29, 45.26 | 4.633 |
| | 210 | Rt frontal medial frontal gyrus | 10 | 17.31, 46.83, -3.16 | 4.595 |
| | 283 | Rt occipital cuneus | | 17.91, -80.95, 19.88 | 4.541 |
| | | Rt occipital precuneus | 31 | 27.66, -75.28, 19.23 | 4.342 |
| | | Rt temporal sub-gyral | | 27.63, -68.68, 23.91 | 4.033 |
| | 111 | Rt limbic parahippocampal gyrus | | 9.94, -42.61, 3.11 | 4.201 |
| **CN>AD** | | | | | |
| | 262457 | Lt limbic anterior cingulate | | -16.28, 33.99, 13.98 | 65535 |
| | | Rt sub-lobar extra-nuclear | | 37.56, -12.31, 24.02 | 65535 |
| | | Rt limbic lobe cingulate gyrus | | 10.96, -30.25, 35.38 | 65535 |
| **MCI>AD** | | | | | |
| | 83376 | Rt sub-lobar extra nuclear | | 37.58, -12.18, 22.68 | 7.572 |
| | | Lt limbic anterior cingulate | | -16.28, 33.99, 13.98 | 7.471 |
| | | Lt frontal lobe sub-gyral | | -28.85, 18.42, 14.99 | 6.415 |

Subject's total intracranial volume (TIV), age, and gender were used as covariates. The white matter volume (WMV) was not significantly different to the following tests: CN<AD, CN<MCI, and MCI<AD.

CN, cognitively normal; MCI, mild cognitive impairment; AD, Alzheimer’s disease; Rt, right; Lt, left; BA, Brodmann area

**Table S4. Results of the voxel-based multiple regression analyses between apparent myelin water fraction (MWF) and ages in all participant groups**

| Group analysis | Cluster size | Cluster location | BA | Talairach coordinates | Z score |
|---|---|---|---|---|---|
| **All Group: (-) Age** | | | | | |
| | 226682 | Lt frontal sub-gyral | | -25.19, -20.51, 42.9 | 6.444 |
| | | Lt parietal sub-gyral | | -25.24, -37.28, 41.31 | 6.423 |
| | | Rt frontal sub-gyral | | 24.85, -18.85, 38.5 | 6.382 |

(-) indicates the negative correlation. We did not find any positive correlation between MWF and age. There were no correlations between MWF and Mini-Mental State Examination (MMSE) scores.

Rt, right; Lt, left; BA, Brodmann area

**Table S5. Results of the voxel-based multiple regression analyses between gray matter volume (GMV) and ages in all participant groups**

| Group analysis | Cluster size | Cluster location | BA | Talairach coordinates | Z score |
|---|---|---|---|---|---|
| **All Group: (-) Age** | | | | | |
| | 143802 | Lt limbic parahippocampal gyrus | 34 | -14.64, -13.45, -20.22 | 6.499 |
| | | Lt temporal fusiform gyrus | 20 | -40.98, -6.19, -21.33 | 6.447 |
| | | Lt temporal sub-gyral | 20 | -42.47, -13.83, -15.32 | 6.416 |
| | 102 | Lt cerebellum posterior cerebellar tonsil | | -6.13, -38.21, -41.34 | 4.153 |
| | 145 | Lt sub-lobar thalamus | | -17.77, -11.98, 8.25 | 4.141 |
| | 293 | Rt frontal superior frontal gyrus | 9 | 18.21, 39.1, 32.61 | 4.075 |
| | | Rt frontal middle frontal gyrus | 8 | 22.28, 30.17, 37.24 | 3.759 |

(-) indicates the negative correlation. We did not find any positive correlation between GMV and age. There were no correlations between GMV and Mini-Mental State Examination (MMSE) scores.

Rt, right; Lt, left; BA, Brodmann area

**Table S6. Results of the voxel-based multiple regression analyses between white matter volume (WMV) and ages in all participant groups**

| Group analysis | Cluster size | Cluster location | BA | Talairach coordinates | Z score |
|---|---|---|---|---|---|
| **All Group: (-) Age** | | | | | |
| | 122989 | Lt limbic anterior cingulate | | -9.18, 37.93, 2.31 | 6.011 |
| | | Lt frontal sub-gyral | | -23.09, 37.74, 4.76 | 5.974 |
| | | Rt frontal sub-gyral | | 26.92, 35.07, 1.3 | 5.890 |
| | 183 | Rt occipital lingual gyrus | | 28.02, -78.12, -9.41 | 4.040 |

(-) indicates the negative correlation. We did not find any positive correlation between WMV and age. There were no correlations between WMV and Mini-Mental State Examination (MMSE) scores.

Rt, right; Lt, left; BA, Brodmann area

**Table S7. Results of a receiver operating characteristic (ROC) curve analysis of MRI measures at the specific brain areas.**

| ROI | MRI measures | CN vs MCI | | | | CN vs AD | | | | MCI vs AD | | | |
|---|---|---|---|---|---|---|---|---|---|---|---|---|---|
| | | SE | SP | AUC | p | SE | SP | AUC | p | SE | SP | AUC | p |
| Corpus Callosum | MWF | 75.00 | 41.38 | 0.517 | 0.8222 | 86.21 | 67.74 | ***0.799*** | <0.0001 | 70.97 | 84.37 | ***0.779*** | <0.0001 |
| | GMV | 81.25 | 48.28 | 0.653 | 0.0329 | 86.21 | 41.94 | 0.641 | 0.0493 | 35.48 | 84.37 | 0.509 | 0.9112 |
| | WMV | 87.50 | 41.38 | 0.627 | 0.0858 | 75.86 | 70.97 | 0.775 | <0.0001 | 48.39 | 90.62 | 0.709 | 0.0018 |
| Cingulate gyrus | MWF | 62.50 | 55.17 | 0.536 | 0.6381 | 68.97 | 74.19 | 0.734 | 0.0004 | 74.19 | 62.50 | 0.700 | 0.0029 |
| | GMV | 56.25 | 72.41 | 0.666 | 0.0177 | 82.76 | 58.06 | 0.729 | 0.0005 | 29.03 | 90.62 | 0.583 | 0.2592 |
| | WMV | 90.62 | 37.93 | 0.660 | 0.0251 | 89.66 | 61.29 | 0.808 | <0.0001 | 61.29 | 81.25 | 0.702 | 0.0030 |
| Cingulum | MWF | 59.38 | 58.62 | 0.543 | 0.5689 | 62.07 | 74.19 | 0.685 | 0.0087 | 35.48 | 93.75 | 0.655 | 0.0268 |
| | GMV | 65.62 | 68.97 | 0.680 | 0.0101 | 86.21 | 64.52 | 0.773 | <0.0001 | 64.52 | 65.62 | 0.653 | 0.0329 |
| | WMV | 75.00 | 55.17 | 0.660 | 0.0253 | 93.10 | 61.29 | 0.820 | <0.0001 | 61.29 | 81.25 | 0.703 | 0.0028 |
| Hippocampus | MWF | 56.25 | 65.52 | 0.572 | 0.3331 | 68.97 | 77.42 | 0.769 | <0.0001 | 61.29 | 71.87 | 0.667 | 0.0154 |
| | GMV | 50.00 | 82.76 | 0.691 | 0.0058 | 86.21 | 80.65 | ***0.883*** | <0.0001 | 61.29 | 84.37 | ***0.771*** | <0.0001 |
| | WMV | 71.87 | 65.52 | 0.659 | 0.0260 | 93.10 | 70.97 | ***0.860*** | <0.0001 | 77.42 | 68.75 | ***0.740*** | 0.0001 |
| Middle temporal gyrus | MWF | 53.13 | 75.86 | 0.597 | 0.1944 | 72.41 | 80.65 | 0.751 | 0.0001 | 77.42 | 68.75 | 0.719 | 0.0009 |
| | GMV | 46.88 | 86.21 | 0.666 | 0.0191 | 82.76 | 80.65 | 0.829 | <0.0001 | 51.61 | 90.62 | 0.720 | 0.0008 |
| | WMV | 81.25 | 58.62 | 0.673 | 0.0171 | 75.86 | 77.42 | 0.816 | <0.0001 | 48.39 | 90.62 | 0.701 | 0.0030 |
| Parahippocampal gyrus | MWF | 75.00 | 48.28 | 0.592 | 0.2183 | 86.21 | 64.52 | 0.771 | <0.0001 | 58.06 | 81.25 | 0.684 | 0.0070 |
| | GMV | 68.75 | 65.52 | 0.687 | 0.0070 | 86.21 | 83.87 | 0.872 | <0.0001 | 77.42 | 71.87 | 0.754 | 0.0001 |
| | WMV | 71.87 | 62.07 | 0.666 | 0.0187 | 75.86 | 77.42 | 0.840 | <0.0001 | 87.10 | 53.13 | 0.734 | 0.0002 |
| Pons | MWF | 90.62 | 27.59 | 0.533 | 0.6614 | 72.41 | 74.19 | 0.733 | 0.0004 | 74.19 | 71.87 | 0.726 | 0.0007 |
| | GMV | 34.38 | 89.66 | 0.572 | 0.3375 | 96.55 | 48.39 | 0.740 | 0.0002 | 80.65 | 53.13 | 0.685 | 0.0065 |
| | WMV | 71.87 | 51.72 | 0.594 | 0.2072 | 75.86 | 61.29 | 0.720 | 0.0010 | 58.06 | 71.87 | 0.641 | 0.0450 |
| Precuneus | MWF | 62.50 | 65.52 | 0.615 | 0.1163 | 93.10 | 54.84 | 0.770 | <0.0001 | 77.42 | 59.38 | 0.679 | 0.0092 |
| | GMV | 84.37 | 48.28 | 0.645 | 0.0431 | 89.66 | 58.06 | 0.746 | 0.0001 | 58.06 | 84.37 | 0.649 | 0.0385 |
| | WMV | 75.00 | 62.07 | 0.693 | 0.0048 | 79.31 | 70.97 | 0.819 | <0.0001 | 74.19 | 56.25 | 0.678 | 0.0093 |
| Thalamus | MWF | 75.00 | 44.83 | 0.550 | 0.5113 | 62.07 | 83.87 | 0.734 | 0.0005 | 83.87 | 56.25 | 0.709 | 0.0018 |
| | GMV | 18.75 | 93.10 | 0.510 | 0.8981 | 93.10 | 38.71 | 0.679 | 0.0105 | 70.97 | 62.50 | 0.672 | 0.0123 |
| | WMV | 46.88 | 82.76 | 0.650 | 0.0385 | 68.97 | 74.19 | 0.722 | 0.0015 | 83.87 | 40.63 | 0.584 | 0.2563 |

CN, cognitively normal; MCI, mild cognitive impairment; AD, Alzheimer's disease; ROI, regions-of-interest; SE, Sensitivity; SP, Specificity; AUC, Area under the ROC curve; MWF, myelin water fraction; GMV, gray matter volume; WMV, white matter volume
